# Seasonal and geographic patterns of gastroschisis in Canada: protective effect of periconceptional sunlight exposure

**DOI:** 10.3389/fendo.2026.1816093

**Published:** 2026-05-05

**Authors:** Shiliang Liu, Shin Jie Yong, Dunjin Chen, Gavin C. Hughes, Claude L. Hughes

**Affiliations:** 1 Behaviours and Environments Division, Centre for Surveillance and Applied Research, Public Health Agency of Canada, Ottawa, ON, Canada; 2 School of Epidemiology and Public Health, Faculty of Medicine, University of Ottawa, Ottawa, ON, Canada; 3 Sir Jeffrey Cheah Sunway Medical School, Faculty of Medical and Life Sciences, Sunway University, Sunway City, Selangor, Malaysia; 4 Department of Obstetrics and Gynecology, Third Affiliated Hospital of Guangzhou Medical University, Guangzhou, China; 5 Eastern Virgina Medical School, Old Dominion University, Norfolk, VA, United States; 6 Division of Reproductive Endocrinology and Infertility, Department of Obstetrics and Gynecology, Duke University Medical Center, Durham, NC, United States; 7 Reproductive Health Center of Excellence, IQVIA, Durham, NC, United States

**Keywords:** causality, gastroschisis, hypovitaminosis D, sunlight exposure, vitamin D

## Abstract

**Background:**

Gastroschisis is a right−sided, full−thickness defect with extra-amnionic herniation of abdominal contents directly into the amniotic cavity. The causes of gastroschisis remain obscure. We hypothesized that limited periconceptional sunlight exposure might lead to insufficient vitamin D during fetal development and play a role in gastroschisis etiology.

**Methods:**

We conducted a population-based cohort study of infants conceived in Canada between April 2006 and March 2020. We used winter months (i.e., November and December) and northern geographic latitudes as indicators of insufficient sunlight exposure, while summer months (July and August) and southern geographic latitudes were considered as reference. We examined the association of conception month and geographic region with infant gastroschisis using log-binomial regression with a Poisson distribution.

**Results:**

Prevalence of gastroschisis varied substantially by month of conception. The rate of gastroschisis for infants conceived in winter months (4.6 per 10 000) was higher than infants conceived in summer months (2.7 per 10 000; p<0.001), yielding an adjusted rate ratio (aRR) of 1.73 [95% confidence interval (CI) 1.39-2.16]. Gastroschisis prevalence increased from 2.9 per 10000 in the lowest latitude (South) to 10.8 in the highest latitude (far North) (aRR 1.89, 95% CI 1.34-2.67). Multivariate regression model showed that seasonal variations in gastroschisis risk varied with rural vs urban residence (aRR 0.68, 95% CI 0.46-0.99; p<0.05).

**Conclusions:**

Our findings indicate that periconceptional exposure to sunlight may protect against gastroschisis in offspring. Some literature inconsistencies about hypovitaminosis D and gastroschisis are clarified by interpreting associations as a non-monotonic (inverted U-shaped) relationship between maternal vitamin D status and the risk of gastroschisis. Further studies are warranted to identify biological mechanisms linking hypovitaminosis D with gastroschisis.

## 1 Introduction

Gastroschisis and omphalocele are distinct congenital abdominal wall defects that differ in anatomic location, embryologic origin, and structural features. Gastroschisis is typically a right−sided, full−thickness defect with extra-amnionic herniation of abdominal contents directly into the amniotic cavity, whereas omphalocele is a midline defect characterized by intra-amnionic herniation of abdominal organs. Gastroschisis prevalence has increased worldwide since the 1960s (1, 2) and varies greatly by geographic region (3–6). While the cause remains largely unknown, the etiology is believed to be multifactorial and primarily non-genetic (2, 7, 8). Currently, young maternal age is the only confirmed risk factor. For example, a 7 times higher risk of gastroschisis is typically observed among women <20 years of age than women aged 25–29 years. Other common risk factors include location of residence, poor nutrition, smoking, and substance abuse (1, 2, 7–9). However, the literature has paid relatively little attention to seasonal variation or how gastroschisis varies with location of residence.

There is growing evidence of geographic variation in the occurrence of gastroschisis. Gastroschisis incidence decreases from north-to-south latitudes in the UK (4, 5), continental Europe (3), and Canada (10), and substantial spatial variation is observed in the US (6) and Canada (11). However, the underlying drivers behind geographic variation in gastroschisis have not been studied. One possible factor may be sunlight exposure and vitamin D levels (12, 13). Numerous studies have highlighted the association between maternal vitamin D deficiency and abnormal fetal growth, preterm birth, small size for gestational age at birth, and reproductive failure (12, 14). Vitamin D is necessary for fetal development, and low levels are associated with birth defects that form before 12 weeks of gestation (12, 13). As gastroschisis develops between the 5^th^ and 10^th^ week of gestation (15), a relationship with maternal vitamin D status is plausible.

Although food may provide small amounts of both vitamin D_3_ (cholecalciferol) and vitamin D_2_ (ergocalciferol), sunlight exposure is by far the major source of vitamin D to the human body. Residential latitude, season and time spent outdoors are main factors determining vitamin D status. As rural people may spend more time outdoors, exposure to sunlight may be greater. Vitamin D related seasonal and residential variations in the occurrence of gastroschisis are therefore plausible.

In this study, we examined whether fetuses conceived in winter months or in residential areas with high latitude and low sun exposure have an increased risk of gastroschisis compared with summer months or southern areas in Canada. Further, we explored whether rural residence could reduce the risk of gastroschisis.

## 2 Materials and methods

### 2.1 Study design and population

We carried out a retrospective cohort study of 3 921889 live births in Canada between April 1, 2006, and March 31, 2020. The data were drawn from the Canadian Institute for Health Information’s Discharge Abstract Database (DAD), which contains records of live-born infants linked to their mothers (16, 17). Stillbirths and terminated pregnancies were not included as variables (e.g., birth date) necessary for the study were unavailable in the DAD.

Additional exclusion criteria included maternal age <14 years or >39 years, gestational age <20 weeks, birth weight <500 grams, and missing information on birth month, gestational age or birth weight (n=686 806). To minimize the potential for confounding, we further excluded multiple pregnancies, infants exposed to maternal tobacco, alcohol, and problematic use of substances (i.e., opioids, cannabinoids, cocaine, and other specified/unspecified drugs) or other mental and behavioral disorders, gestational diabetes, obesity and hypothyroidism based on ICD-10 codes documented in maternal records (n=448 405) (10, 18, 19). A total of 2 788678 infants were included in the main analysis.

### 2.2 Exposures

Month of conception and latitude of maternal residence were used as proxies for sunlight exposure and vitamin D status around the time of conception and in early fetal development. The month of conception, a primary exposure measure, was determined using the birth month and the number of completed weeks of gestation at birth, using the formula:


Month of conception=birth month–(((gestational weeks x 7)–(14+3))/30.4)

where (i) 7 is used to convert gestational weeks to days; (ii) 14 + 3 indicates two weeks (i.e., 14 days) of the last menstrual period (LMP) plus one incomplete week (i.e., average of 3 days); and (iii) 30.4 is an approximation to convert days to months (365 days divided by 12 months).

**Figure 1:**
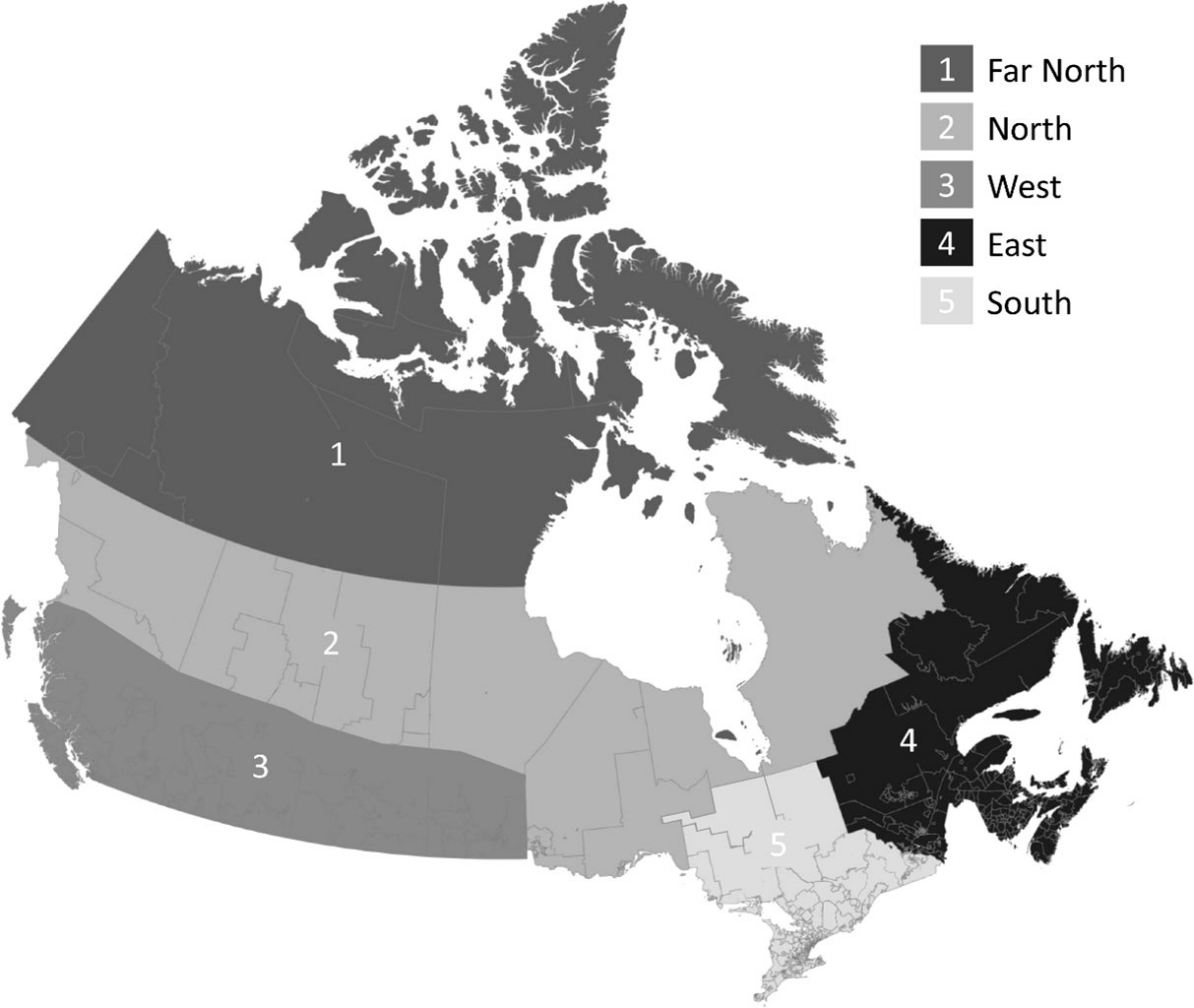
Map of Canadian geographic regions.

Pre-defined sub-regions based on postal codes of maternal residence (10, 20) were used to classify infants into five geographic regions with differential latitudes reflecting degree of sunlight exposure and vitamin D status (21–23): (i) *Far North* consisting of the three Territories; (ii) *North* including northern areas of British Columbia, Alberta, Saskatchewan, Manitoba and Ontario; (iii) *West* consisting of southern parts of British Columbia, Alberta, Manitoba and Saskatchewan; (iv) *East* consisting of Newfoundland and Labrador, Nova Scotia, Prince Edward Island and New Brunswick; and (v) *South* including western, central and eastern Ontario (**Figure 1**).

### 2.3 Outcome and covariates

Gastroschisis was collected from the live birth record using the International Classification of Diseases codes (ICD-10 Q793). Gastroschisis is usually detected prenatally and confirmed at birth.

Maternal characteristics and other covariates studied included age, parity, infant sex, and maternal chronic conditions or illnesses consisting of pre-existing diabetes, lupus, epilepsy, migraine, hyperthyroidism and thyroiditis identified through ICD-10 codes (10). Rural or urban maternal residence was identified using the forward sortation area of residential postal code (24). We included maternal residence to potentially account for variations in accessibility to prenatal screening/diagnosis and subsequent termination, and because this variable is a partial surrogate for socio-economic status (SES) (25).

### 2.4 Statistical analysis

We examined gastroschisis prevalence in each month of conception (**Supplementary Tables 1**, **2**). We used July and August (summer season), months with the highest sunlight exposure or vitamin D status, as the reference. We compared 5 other two-month periods, including November-December, to this reference. We did find the well-known maternal age-related pattern of increased risk in younger gravidas but lowered risk in older gravidas (**Table 1**).

**Table 1:** Association of conception month and residential latitude with gastroschisis in offspring.

Characteristic	No. of mother (% of total N= 2788 678)	Cases (n=1121; number, rate per 10 000)	Unadjusted rate ratio & 95% confidence intervals	Adjusted rate ratio & 95% confidence intervals ^a^	Adjusted rate ratio & 95% confidence intervals ^c^
Conception month					
Winter					
Nov - Dec	498 565 (17.9)	230 (4.6)	1.72 (1.38 – 2.14)	1.73 (1.39 - 2.16)	1.95 (1.51 - 2.52)
Summer					
Jul - Aug	455 275 (16.3)	122 (2.7)	1.00 (reference)	1.00 (reference)	1.00 (reference)
Other months					
Jan - Feb	483 745 (17.3)	203 (4.2)	1.57 (1.25 – 1.96)	1.58 (1.26 – 1.97)	1.77 (1.36 – 2.31)
Mar - Apr	437 173 (15.7)	163 (3.7)	1.39 (1.10 – 1.76)	1.38 (1.09 – 1.75)	1.55 (1.18 – 2.04)
May - Jun	433 717 (15.6)	195 (4.5)	1.68 (1.34 – 2.10)	1.67 (1.33 – 2.09)	1.88 (1.44 - 2.45)
Sep - Oct	480 203 (17.2)	208 (4.3)	1.62 (1.29 – 2.02)	1.63 (1.31 – 2.04)	1.84 (1.41 - 2.39)
Geographic region					
Far North	37 037 (1.3)	40 (10.8)	3.71 (2.67 - 5.14)	1.89 (1.34 – 2.67)	1.89 (1.34 – 2.67)
North	176 545 (6.3)	135 (7.6)	2.63 (2.15 - 3.53)	1.52 (1.23 – 1.89)	1.52 (1.23 – 1.89)
West	1 127 872 (40.5)	487 (4.3)	1.48 (1.29 - 1.70)	1.28 (1.11 – 1.49)	1.29 (1.11 – 1.49)
East	221 276 (7.9)	102 (4.6)	1.58 (1.27 - 1.97)	1.16 (0.88 - 1.53)	1.16 (0.88 – 1.53)
South	1 225 948 (44.0)	357 (2.9)	1.00 (reference)	1.00 (reference)	1.00 (reference)
Rural residence					
Yes	519 127 (18.6)	311 (6.0)	1.68 (1.47 - 1.91)	1.07 (0.93 – 1.24)	1.50 (1.04 - 2.17)
No	2 269 551 (81.4)	810 (3.6)	1.00 (reference)	1.00 (reference)	1.00 (reference)
Interaction of conception month with rural residence ^b^			1.71 (1.49 – 1.96)^†^		0.68 (0.46 – 0.99)^‡^
Maternal age (year)					
<20	128 324 (4.6)	278 (21.7)	8.08 (6.80 - 9.50)	7.03 (5.90 - 8.38)	7.03 (5.90 – 8.38)
20-24	491 088(17.6)	460 (9.4)	3.48 (2.99 - 4.04)	3.34 (2.88 - 3.88)	3.34 (2.87 – 3.88)
25-29	1 009 602 (36.2)	272 (2.7)	1.00 (reference)	1.00 (reference)	1.00 (reference)
30-34	1 159 664 (41.6)	111 (1.0)	0.36 (0.28 - 0.44)	0.37 (0.30 - 0.46)	0.37 (0.30 - 0.46)
Parity					
1^st^ child	1 062 725 (38.1)	587 (5.5)	2.30 (1.94 - 2.71)	1.54 (1.30 - 1.83)	1.54 (1.30 - 1.83)
2^nd^ child	752 901 (27.0)	181 (2.4)	1.00 (reference)	1.00 (reference)	1.00 (reference)
≥3^rd^ child	414 208 (14.9)	120 (2.9)	1.21 (0.96 - 1.52)	1.41 (1.12 - 1.78)	1.41 (1.12 - 1.78)
Missing	558 844 (20.0)	233 (4.2)	1.73 (1.43 - 2.11)	1.40 (1.12 - 1.75)	1.40 (1.12 - 1.75)
Infant sex					
Male	1 429 000 (51.2)	569 (4.0)	0.98 (0.87 - 1.10)	0.98 (0.87 - 1.10)	0.98 (0.87 - 1.10
Female	1 359 678 (48.8)	552 (4.1)	1.00 (reference)	1.00 (reference)	1.00 (reference)
Chronic illness ^c^					
Yes	271 02 (1.0)	10 (3.7)	1.03 (0.55 - 1.93)	1.03 (0.55 - 1.93)	1.03 (0.55 - 1.93)
No	2 761 576 (99.0)	1 111 (4.0)	1.00 (reference)	1.00 (reference)	1.00 (reference)

^a^ Adjusted for variables in the first column of the table. ^b^ Including pre-existing diabetes, lupus, epilepsy, migraine, hyperthyroidism or thyroiditis among mothers; ^c^ Interaction term: rural residence (yes = 1, no = 0), and month of conception (July-August = 0, other months =1). ^d^ Adjusted for all variables, plus the interaction. ^†^ p <0.001. ^‡^ p =0.0.

A log-binomial regression model with a Poisson distribution was used to model the association of gastroschisis with season of conception. Univariable and multivariable rate ratios with 95% confidence intervals were estimated, with the latter adjusted for geographic region, maternal age, rural residence and additional covariates such as parity, infant sex, and chronic illness.

As we postulated that vitamin D status may be higher for women living in rural areas (26–28), further analysis was performed to assess whether sun exposure effect of residential latitudes was moderated by rural (versus urban) residence. We added an interaction term for month of conception with rural residence to the final multivariate model.

### 2.5 Negative control approach and ethics statement

Omphalocele (ICD-10 Q792) is a congenital abdominal wall defect that is causally distinct from gastroschisis and frequently associated with aneuploidy (3, 7). There is neither reported North-to-South decreasing gradient nor known mechanism linking maternal vitamin D levels or lifestyle factors with omphalocele in the literature. To assess whether the association observed for gastroschisis is due to uncontrolled confounding (29), we applied the same participant inclusion criteria and repeated all the analyses using omphalocele as negative control outcome (**Table 2**).

**Table 2:** Association of conception month and residential latitude with omphalocele in offspring.

Characteristic	Singleton deliveries (% of total N= 2 788 678)	Cases (n=365; Number, rate per 10 000)	Rate ratio (RR) 95% Confidence intervals)		
			Unadjusted	Adjusted ^a^	Adjusted ^d^
Conception month					
Winter					
November - December	498 565 (17.9)	62 (1.24)	0.93 (0.65 – 1.32)	0.93 (0.65 – 1.32)	0.93 (0.65 - 1.32)
Summer					
July-August	455 275 (16.3)	61 (1.34)	1.00 (reference)	1.00 (reference)	1.00 (reference)
Other months					
January - February	483 745 (17.3)	75 (1.55)	1.16 (0.98 – 1.62)	1.16 (0.82 – 1.62)	1.16 (0.82 - 1.62)
March - April	437 173 (15.7)	49 (1.12)	0.84 (0.57 – 1.22)	0.83 (0.57 - 1.22)	0.83 (0.57 - 1.22)
May - June	433 717 (15.6)	63 (1.45)	1.08 (0.76 - 1.54)	1.08 (0.76 - 1.54)	1.08 (0.76 - 1.54)
September - October	480 203 (17.2)	55 (1.15)	0.86 (0.55 – 1.23)	0.86 (0.59 – 1.23)	0.86 (0.59 - 1.23)
Geographic region					
Far North	37 037 (1.3)	10 (2.70)	2.02 (1.07 – 3.82)	2.35 (1.22 – 4.54)	1.05 (0.20 - 5.49)
North	176 545 (6.3)	25 (1.42)	1.06 (0.70 – 1.61)	1.19 (0.76 - 1.86)	0.64 (0.19 - 2.22)
West	1 127 872 (40.5)	149 (1.32)	0.99 (0.79 - 1.23)	1.03 (0.80 - 1.31)	0.67 (0.29 - 1.54)
East	2 21 276 (7.9)	17 (0.77)	0.57 (0.35 – 0.95)	0.62 (0.35 - 1.10)	0.50 (0.25 - 1.003)
South	1 225 948 (44.0)	164 (1.34)	1.00 (reference)	1.00 (reference)	1.00 (reference)
Rural residence					
Yes	519 127 (18.6)	62 (1.19)	0.90 (0.68 – 1.18)	0.88 (0.65 – 1.18)	0.88 (0.66 - 1.19)
No	2 269 551 (81.4)	303 (1.34)	1.00 (reference)	1.00 (reference)	1.00 (reference)
Interaction of conception season with rural residence ^b^			0.999 (0.996 - 1.002) ^†^		0.992 (0.979 - 1.007) ^‡^
Maternal age (year)					
<20	128 324 (4.6)	16 (1.25)	1.04 (0.62 – 1.75)	0.94 (0.55 – 1.59)	0.74 (0.37 – 1.48)
20-24	491 088 (17.6)	67 (1.36)	1.14 (0.85 – 1.53)	1.11 (0.82 – 1.50)	0.97 (0.66 - 1.44)
25-29	1 009 602 (36.2)	121 (1.20)	1.00 (reference)	1.00 (reference)	1.00
30-34	1 159 664 (41.6)	161 (1.39)	1.16 (0.92 – 1.47)	1.18 (0.93 – 1.50)	1.35 (0.96 - 1.91)
Parity					
1^st^ child	1 062 725 (38.1)	162 (1.52)	1.02 (0.91 – 1.51)	1.19 (0.93 - 1.54)	1.18 (0.92 - 1.53)
2^nd^ child	752 901 (27.0)	98 (1.30)	1.00 (reference)	1.00 (reference)	1.00 (reference)
≥3^rd^ child	414 208 (14.9)	42 (1.01)	0.78 (0.94 - 1.18)	0.76 (0.53 – 1.10)	1.31 (0.53 - 1.10)
Missing data	558 844 (20.0)	63 (1.13)	0.87 (0.631 – 1.19)	1.01 (0.70 - 1.45)	1.00 (0.69 - 1.44)
Infant sex					
Male	1 429 000 (51.2)	238 (1.27)	0.95 (0.77 – 1.16)	0.95 (0.77 – 1.16)	0.95 (0.77 - 1.16)
Female	1 359 678 (48.8)	228 (1.35)	1.00 (reference)	1.00 (reference)	1.00
Chronic illness ^c^					
Yes	27 102 (1.0)	7 (2.58)	1.99 (0.94 – 4.21)	2.00 (0.95 – 4.24)	2.00 (0.95 - 4.24)
No	2761 576 (99.0)	358 (1.30)	1.00 (reference)	1.00 (reference)	1.00 (reference)

^a^ Adjusted for all variables in the first column of the table. ^b^ Interaction term: rural residence (yes = 1, no = 0), and month of conception (July-August = 0, other months =1). ^c^ Including pre-existing diabetes, lupus, epilepsy, migraine, hyperthyroidism or thyroiditis among mothers. ^d^ Adjusted for all variables, plus the interaction. ^†^ p =0.508. ^‡^ p =0.296.

This study was carried out under the surveillance mandate of the Public Health Agency of Canada, and ethics approval was not required.

## 3 Results

### 3.1 Population prevalence

A total of 1121 gastroschisis cases were identified from 2 788678 mother-infant dyads eligible for the study, yielding an overall prevalence of 4.02 [95% confidence interval (CI) 3.79-4.26] per 10000 singleton live births between 2006 and 2020.

### 3.2 Seasonality of gastroschisis

Gastroschisis rates for infants conceived in November (4.64, 95% CI 3.83-5.56) per 10000 and December (4.63, 95% CI 3.82-5.55) were significantly higher than those conceived in July (3.00, 95% CI 2.33-3.80) and August (2.36, 95% CI 1.77-3.08) per 10000 singleton infants, respectively. However, rates of gastroschisis appeared to vary slightly by month of conception from January through June, and in September-October: from a low of 3.66 (95% CI 2.90–4.56) per 10000 in April to a high of 4.53 (95% CI 3.71-5.47) in February. Compared with the summer months (July-August), infants conceived in winter months (September to June) had the highest risk of gastroschisis: rate ratio (RR) 1.72 (95% CI 1.38-2.14). See **Supplementary Tables 1** and **2**.

### 3.3 Geographic latitudes and rural versus urban living

Gastroschisis prevalence increased from the lowest to the highest latitude, from 2.9 per 10000 in South to 10.8 in far North. Compared with South, the rate ratios of gastroschisis showed an approximately 4-fold increase (RR 3.7, 95% CI 2.7-5.1) and 2.6-fold increase (RR 2.6, 95% CI 2.2-3.5) in far North and North, respectively (**Table 1**).

Interaction term of rural residence with conception month was significantly associated with increased risk of gastroschisis (RR 1.71, 95% CI 1.49-1.96). Adjustments for all covariates showed little change to the seasonal variations while the rate ratio for rural residence changed to a non-significant increase (aRR 1.07, 95% CI 0.93-1.24). Although the south-to-north increases diminished significantly, there remained a significant increase in the risk of gastroschisis from South to the far North, by nearly 2-fold (aRR 1.89, 95% CI 1.34-2.67). As such, rate ratio for East [aRR 1.58 (95% CI 1.27-1.97)] was substantially attenuated to aRR 1.16 (95% CI 0.88-1.53) compared with South (**Table 1**).

Secondary analysis showed that interaction of rural (vs urban) residence with conception months was negatively associated with the seasonal variations (aRR 0.69, 95% CI 0.46-0.99). Seasonal effect of sunlight exposure on the risk of gastroschisis may vary with residential location or latitudes. For example, risk of gastroschisis could be moderated to aRR 1.95 (95% CI 1.51–2.52) for winter vs summer months. However, the addition of interaction did not lead to change to the rate ratios for other characteristics compared with the multivariate regression model without the term. Instead, rural residence retained to be positively associated with gastroschisis (aRR 1.50, 95% CI 1.04–2.17), possibly reflecting lower accessibility to prenatal screening/diagnosis and subsequent termination of pregnancies due to congenital anomalies in rural areas or an independent association with lower socioeconomic status (**Table 1**).

### 3.4 Omphalocele as a negative control

Analyses of association between conception month and risk of omphalocele showed no seasonal variation (**Table 2**). Winter months were not associated with risk of omphalocele compared with summer months (aRR 0.93, 95% CI 0.65-1.32). Compared with South, far North alone was observed to be associated with increased risk of omphalocele (aRR 2.35, 95% CI 1.22–4.54) but turned out to be non-significant (aRR 1.05, 95% CI 0.20-5.49) after adding an interaction of rural residence with conception month. These changes indicated that the increased risk of omphalocele in far North seemed largely restricted to rural area. Additionally, risk of omphalocele was not significantly associated with conception month. However, despite its non-significance, change to the interaction term suggested the possibility of lower prenatal screening and pregnancy termination in Northern Canada (**Table 2**).

## 4 Discussion

### 4.1 Primary findings

In this Canada-wide population-based study, we found that infants conceived in months with less sunlight (i.e., November and December) were at a significantly increased risk of gastroschisis than infants conceived in months with longer sun exposure (i.e., July and August). The association between season of conception and gastroschisis remained after adjustments for multiple potential confounders. The risk of gastroschisis and its seasonal variations might be greater in northern vs southern/eastern areas. Our findings provide evidence supporting the hypothesis that increased periconceptional exposure to sunlight potentially implicating vitamin D, can protect against gastroschisis during fetal development. Findings from the negative control outcome (i.e., omphalocele) approach support the association.

Our data show changes in rates of gastroschisis associated with season of conception. Consideration of causality could plausibly be related to either a) the constellation of multiple other metabolic seasonal changes associated with photoperiod length as a stressor, b) photoperiod-related changes in maternal vitamin D during early embryogenesis, or c) a combination of these alternatives, potentially interacting with other known risk factors for gastroschisis such as younger maternal age, substance use, levels of stress, and possibly even unrecognized maternal hypothyroidism (30).

As recently presented in a comprehensive review by Regmi et al. (31), photoperiod profoundly affects metabolism and substantial alterations in photoperiod may adversely affect metabolic health. The extent to which more moderated changes in photoperiod as occurs with seasonality in higher latitudes may act as a maternal metabolic stressor is not thoroughly understood. Nonetheless, it is well understood that intrinsically photosensitive retinal ganglion cells encode luminance (32) information to the suprachiasmatic nuclei (SCN) which transmit neuronal and chemical signals that in turn entrain peripheral circadian clocks and synchronize multiple neuroendocrine, CNS and autonomic nervous system rhythms. Collectively these actions profoundly influence functions and metabolism of other organs. Changes in photoperiod can cause transcriptome changes in the SCN and in turn modulate or disrupt the circadian pattern in these various downstream targets. For our purpose of considering potential effects of photoperiod on risk of gastroschisis, even modest dysfunction such as disordering of sleep-wake cycles or patterns of hormone secretion and metabolism, could, in effect, be a stressor that incrementally increases risk of this adverse developmental outcome.

The primary role of vitamin D is to maintain calcium and phosphorus homeostasis to preserve bone health. Vitamin D is involved in skeletal development (13) and various non-skeletal disorders, such as allergies and impaired neurodevelopment (33). Plasma serum vitamin D levels closely correlate with sun exposure and seasonality, with more prevalent vitamin D deficiency (<50 nmol/L) during the colder seasons (21). Moreover, it is reported that a third of Canadians are vitamin D deficient, and less than 10% have vitamin D levels above 100 nmol/L (23). Furthermore, it has been shown that exposure to winter sunlight in Northern Canada does not promote previtamin D_3_ synthesis in human skin (21). Therefore, widespread vitamin D insufficiency could plausibly explain the increased risk of gastroschisis among infants conceived in winter months and among mothers in Northern Canada.

A number of relevant studies directly or indirectly support our findings and the vitamin D deficiency hypothesis of gastroschisis. About 40% of Canadians have serum vitamin D levels below 50nmol/L in winter, compared with 25% in the summer (23). Greene-Finestone et al. (27) analyzed data from seven cities across Canada in the randomly selected, population-based Canadian Multicenter Osteoporosis Study (CaMos), and reported that female participants had the highest vitamin D status in July and August. Paranjothy et al. (9) found that higher intake of fruits and vegetables during the first trimester, longer duration of folic acid supplementation and higher body fat percentage are associated with reduced risk of fetal gastroschisis. Higher body fat is reported to be positively associated with vitamin D status (26) (23). Feldkamp et al. (34) found that increasing diet quality was associated with a reduced risk of gastroschisis only among Hispanic and foreign-born women. Vitamin D insufficiency/deficiency is known to be higher for Hispanic and other ethnic minority groups than white women (26). On one side, several studies and a meta-analysis have found an association between lower vitamin D levels early in gestation and higher risk of gestational diabetes mellitus (35, 36). On the other side, gestational diabetes was inversely (risk ratios range from 0.3-0.7) associated with gastroschisis in two large population-based studies (10, 18). Because gastroschisis develops in the first trimester and gestational diabetes is typically mild and undetectable until later in pregnancy, these findings support our hypothesis that pregnant women with highly insufficient vitamin D status may be associated with increased risk of gastroschisis. However, such association might be confounded in some observational studies by maternal conditions such as gestational diabetes and depression (37, 38). Interestingly, unlike most cross-sectional studies, a meta-analysis of longitudinal cohort studies measuring vitamin D levels at baseline in non-depressed individuals revealed a significantly increased hazard ratio of depression (HR 2.21, 95% CI 1.40-3.49) for the lowest vs. highest vitamin D categories (39).

### 4.2 Reconceptualization of hypovitaminosis D and risk of gastroschisis

Various demographic, lifestyle, socioeconomic, environmental and medical comorbidities are factors that have been implicated in the risk of gastroschisis. Over many years, a number of explicatory biological hypotheses have been advanced but no single one of these various proposed mechanisms seems to adequately incorporate the potential influence of the multiple risk factors associated with the occurrence of this disorder. This lack of consilience implies that we should expect there to be more than one causal pathway or multiple points of influence of such a pathway that could in concert lead to a periconception and early embryonic high-risk state in which gastroschisis risk is increased. We hypothesize that limited periconceptional sunlight exposure might lead to insufficient vitamin D during fetal development and play a role in gastroschisis etiology.

Vitamin D deficiency or insufficiency may adversely affect expression of some structural proteins and developmental signaling molecules that are also affected by stress as was recently reported in a drug-induced model of maternal stress Bablok et al. (40). As mentioned previously, there is a common hypothesis that gastroschisis is by some means caused by a failure in vascular or muscular development. Vitamin D has been shown in multiple studies to increase levels of fibronectin (41–43). Given the importance of fibronectin for the development of blood vessels and musculature, and the positive effect of vitamin D on fibronectin levels, there is a clear argument to be made for the role of vitamin D in lowering gastroschisis risk through supporting fibronectin expression.

One of the past proposed mechanistic hypotheses for the occurrence of gastroschisis is a disordering of mechanosensitivity and mechanotransduction signaling during early embryonic development of the abdominal wall (44), with this concept potentially relevant either to the abdominal wall or the abdominal vasculature. None of the proposed candidate genes for abdominal wall development seem to be regulated by vitamin D, but some of the integrins that are expressed in smooth muscle (as in the vasculature) are, specifically integrin subunit alpha V (45, 46), and integrin subunit beta 1 (47). If vitamin D deficiency/insufficiency is having an adverse effect via diminished expression of these two integrins in smooth muscle, this would imply a role via the vascular compromise hypothesis rather than the abdominal wall musculature hypothesis.

In summary, we agree with Bablok et al. (40) and others that stress is a major factor influencing the risk of developing gastroschisis. However, we propose that superimposed on that variable background of stress are some definable factors that heighten risk of gastroschisis, and these include maternal depression with hypothyroidism (30, 48) potentially via diminished action upon a non-genomic thyroid hormone receptor within alphaVbeta3 integrin and now also possibly via vitamin D deficiency/insufficiency.

Characterizing these more explicit factors that may mediate the risk of gastroschisis such as vitamin D deficiency/insufficiency logically presents an opportunity for public health intervention(s) to reduce the risk of this devastating neonatal outcome. Vitamin D deficiency/insufficiency is extremely prevalent and heavily dependent on the season and the region where one lives. Additionally, in mechanistic terms, increased risk of gastroschisis may in part be explained by vitamin D deficiency/insufficiency since that nutritional state would negatively affect a number of the same structural proteins and developmental signaling molecules that could plausibly mediate the occurrence of gastroschisis.

As argued by Chuaire-Noack (49), if a common pathway for multiple stressors acting as risk factors for gastroschisis is via oxidative stress in the embryonic tissues, then the evidence that vitamin D should protect against oxidative stress injury by increasing levels of antioxidant enzymes (50), implies that exposure to maternal stressors in the presence of low vitamin D should amplify the stress-mediated injury and exacerbate the risk of gastroschisis when these two exposures co-occur.

**Figure 2:**
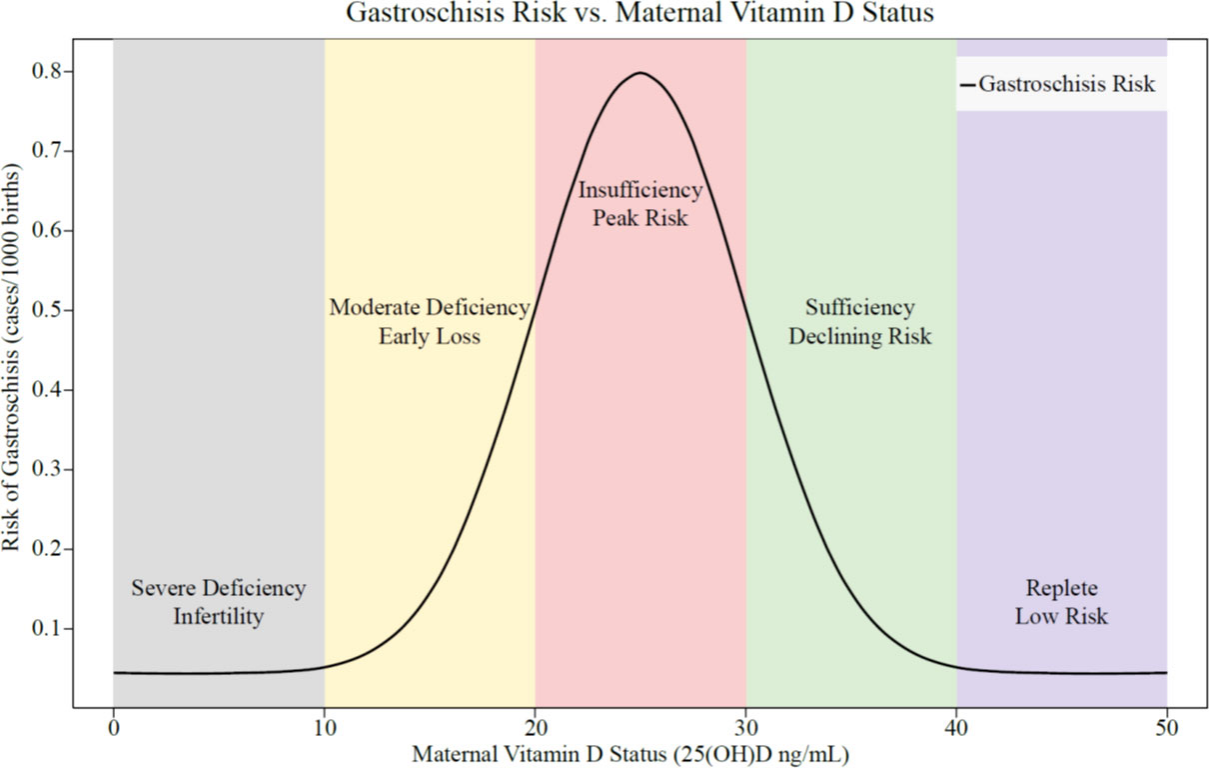
Illustrative conceptual figure of gastroschisis risk vs. maternal vitamin D status.

Using the designations for vitamin D concentrations of deficient <12 ng/mL (or <30 nmol/L), insufficient 12−30 ng/mL (or 30−77 nmol/L), and sufficient >30 ng/mL (or >77 nmol/L), literature searches relating vitamin D deficiency or vitamin D insufficiency to the occurrence of gastroschisis do not yield a substantial weight of evidence for an association. Nonetheless, the recent study by Adrien et al. (51) using dietary vitamin D calculated from food frequency questionnaires, National Oceanic and Atmospheric Administration Weather Service to assign UV indices based on location and estimated date of conception and seasons of conception categorized as fall/winter or spring/summer, lower pre-pregnancy dietary vitamin D intake was associated with increased risks of several congenital anomalies including gastroschisis. We think that the lack of clarity about hypovitaminosis D as a risk factor for gastroschisis relates to the fact that the developmental toxicology outcome of this congenital anomaly is entangled with reproductive toxicology effects that partially obscure the adverse developmental outcome. Accordingly, our key interpretive concept is that maternal vitamin D levels influence early pregnancy developmental outcomes in a non-monotonic fashion as shown in **Figure 2**, which was generated by Microsoft 365 Copilot from analysis of a limited number of pertinent references selected by the authors (51–57).

We propose that:

- Severe vitamin D deficiency in women leads to infertility, with the number of detectable pregnancies being profoundly reduced by several anti-fertility effects of very low vitamin D bioactivity. Multiple subfertility effects of low vitamin D include diminished ovulation, ovarian follicular development, steroidogenic function, and oocyte maturation (58); altered genomic imprinting through epigenetic mechanisms in oocytes with reduced fertilization and developmental competence of oocytes (59); untoward effects on ovarian reserve, ovarian steroidogenesis, follicular development and the endometrium (60); and potentially negative impact on folliculogenesis, decreased pre-antral, antral and mature follicle numbers, and in the case of women with PCOS, elevated occurrence of atretic and cystic follicles, insufficient corpora lutea formation and arrested folliculogenesis (56).
- Moderate deficiency/insufficiency allows better fecundity but there is an increased risk of early embryonic loss (biochemical pregnancies, spontaneous abortions) (54, 55, 61–65), effectively an embryocidal outcome for some increased number of the possibly adversely affected embryos.
- Near the threshold between insufficiency and sufficiency, pregnancies survive but are at increased risk for multiple developmental defects such as gastroschisis.
- With sufficiency and into the normal range, the risk of developmental defects including gastroschisis will decline.
- The relationship between vitamin D status and risk will be non-monotonic (inverted U-shaped): risk is low at both extremes (severe deficiency, no or very few pregnancies; sufficient to replete, low risk of anomalies) but with a modest peak at intermediate/threshold levels. This pattern will be a case of a classic non-monotonic, inverted U-shaped dose-response, as described in the hormesis and endocrine disruption literature (52, 53). The epidemiologic data from the National Birth Defects Prevention Study (NBDPS) demonstrate that low maternal dietary vitamin D intake prior to pregnancy is associated with a significantly increased risk of gastroschisis, as well as other congenital anomalies such as anencephaly, diaphragmatic hernia, and septal defects (51). This pattern is consistent with the concept of non-monotonic dose-response relationships.

### 4.3 Implications for prevention and research

This non-monotonic model of hypovitaminosis D has important implications for both clinical practice and research.

- Screening and supplementation: It illustrates the need for assessment and correction of vitamin D deficiency in women of reproductive age, especially those planning pregnancy.
- Public health messaging: It suggests that across the spectrum of deficiency and insufficiency, hypovitaminosis D may have distinct and clinically meaningful consequences, ranging from infertility to increased risk of miscarriage and congenital anomalies.
- Research directions: Future studies should further delineate the dose-response relationship between vitamin D and gastroschisis and explore the mechanisms by which hypovitaminosis D and other co-factors may interact with vitamin D status to influence fetal development.

### 4.4 Strengths and limitations

To our knowledge, this is the first study testing the hypothesis of the role of sunlight exposure in the etiology of gastroschisis. The strengths of our study include use of data from a vast geography with differential latitudes (e.g., far North, North, West, and South/East), and the distinctive seasonal variation. Information on postal codes for maternal residence is complete and accurate and has been successfully used to define residential location (i.e., rural or urban areas) and latitudes (10, 20). Our use of negative control outcome approach to rule out uncontrolled confounding, and misclassification of the two abdominal wall defects is another strength of the study. Absence of an association between exposure of study and omphalocele supports that substantial residual or unmeasured confounding may be unlikely. However, remaining variations in socioeconomic status, maternal stress levels, education, diet, ethnicity, vitamin D supplementation, etc., could have confounded our analyses. In addition, women in southern/eastern areas and summer months may have better diet quality with higher folic acid and other nutrients with seasonality and regions possibly being proxies of exposures other than vitamin D. While pregnancy terminations or stillbirths due to gastroschisis have become uncommon in Canada (10), absence of the data may impact the temporal prevalence and regional variation (16). Missing information on the gestational days in addition to the completed weeks might have led to misclassification of conception month. However, such non-differential misclassifications should be extremely low or minimal, if any, such bias could generally work toward the null.

## 5 Conclusions

In conclusion, given the seasonal and geographic variations in gastroschisis occurrence in Canada and many other parts of the world, particularly in the Northern hemisphere, we speculated that northern regions with low sunlight exposure may be associated with an increased risk of gastroschisis. Our study has found that conceptions occurring in shorter sunlight months or higher latitudes are at increased risk of gastroschisis compared to longer sunlight months of winter and/or lower latitudes. Our findings that risk of gastroschisis is associated with seasonal and regional factors indicative of low sunlight exposure suggest that vitamin D (i.e., vitamin D_3_, primarily provided by sun exposure) could play a role in the etiology of gastroschisis. Further studies are warranted to confirm the finding and to identify biological mechanisms linking vitamin D with gastroschisis.

Additionally, the non-monotonic, inverted U-shaped risk curve for hypovitaminosis D and gastroschisis provides a coherent framework for understanding the complex interplay between vitamin D status, reproductive success, and developmental outcomes including the risk of this serious congenital anomaly. This concept, supported by mechanistic, clinical, and epidemiologic evidence, illustrates how the risk of gastroschisis (and possibly other adverse developmental outcomes) is highest across the threshold between vitamin D insufficiency and sufficiency. It highlights the need for targeted interventions to ensure adequate vitamin D status before and during pregnancy and suggests that hypovitaminosis D in the preimplantation and early embryonic stage is a critical window of vulnerability for gastroschisis.

## Data Availability

The raw data supporting the conclusions of this article will be made available by the authors, without undue reservation.

## Supplementary material

Supplementary Table 1 Variation in the risk of gastroschisis by conception month in ‘initial’ mother-infant dyads, Canada (excluding Quebec), 2006 to 2020.

Supplementary Table 2 Variation in the risk of gastroschisis by pre-conception month in mother-infant dyads of the study, Canada (excluding Quebec), 2006 to 2020
