# Supplementary material for: Seasonal and geographic patterns of gastroschisis in Canada: protective effect of periconceptional sunlight exposure
Source: Front Endocrinol (Lausanne). 2026 May 5;17:1816093. doi: 10.3389/fendo.2026.1816093 (PMC13183557; doi:10.3389/fendo.2026.1816093)
Supplement: Supplementary Table 1 — Variation in the risk of gastroschisis by conception month in ‘initial’ mother-infant dyads, Canada (excluding Quebec), 2006 to 2020. [file Table1.docx]

Supplementary Table 1. Variation in the risk of gastroschisis by conception month in 'initial' mother-infant dyads, Canada (excluding Quebec), 2006 to 2020

| Pre-conception month | No. of pregnancies | Gastroschisis birth | Rate per 10 000 live births &  95% confidence intervals | Rate ratio &  95% confidence intervals |
| --- | --- | --- | --- | --- |
| January | 333 163 | 125 | 3.75 (3.12 – 4.47) | 1.90 (1.40 – 2.57) |
| February | 313 626 | 96 | 3.06 (2.48 – 3.74) | 1.55 (1.13 - 2.12) |
| March | 306 858 | 89 | 2.90 (2.33 – 3.57) | 1.47 (1.06 – 2.02) |
| April | 316 213 | 108 | 3.42 (2.80 – 4.12) | 1.73 (1.26 - 2.35) |
| May | 297 642 | 109 | 3.66 (3.01 – 4.42) | 1.85 (1.36 - 2.52) |
| June | 318 817 | 73 | 2.29 (1.79 – 2.88) | 1.16 (0.83 – 1.62) |
| July | 318 221 | 63 | 1.98 (1.52 – 2.53) | 1.00 |
| August | 334 169 | 109 | 3.26 (2.68 – 3.93) | 1.65 (1.21 - 2.25) |
| September | 331 819 | 120 | 3.62 (3.00 – 4.32) | 1.83 (1.35 - 2.48) |
| October | 347 953 | 131 | 3.76 (3.15 – 4.47) | 1.90 (1.41 - 2.57) |
| November | 337 919 | 127 | 3.65 (3.04 – 4.34) | 1.84 (1.36 - 2.49) |
| December | 346 058 | 108 | 3.12 (2.56 – 3.77) | 1.58 (1.16 - 2.15) |
| Total | 3 912 458 | 1 258 | 3.22 (3.04 – 3.40) |  |
