# Supplementary material for: Seasonal and geographic patterns of gastroschisis in Canada: protective effect of periconceptional sunlight exposure
Source: Front Endocrinol (Lausanne). 2026 May 5;17:1816093. doi: 10.3389/fendo.2026.1816093 (PMC13183557; doi:10.3389/fendo.2026.1816093)
Supplement: Supplementary Table 2 — Variation in the risk of gastroschisis by pre-conception month in mother-infant dyads of the study, Canada (excluding Quebec), 2006 to 2020 [file Table2.docx]

Supplementary Table 2. Variation in the risk of gastroschisis by pre-conception month in mother-infant dyads of the study, Canada (excluding Quebec), 2006 to 2020

| Pre-conception month | Live singleton deliveries | Gastroschisis birth | Rate per 10 000 live births &  95% confidence intervals | Rate ratio &  95% confidence intervals |
| --- | --- | --- | --- | --- |
| January | 285 054 | 109 | 3.82 (3.14 – 4.61) | 1.84 (1.34 - 2.54) |
| February | 267 282 | 85 | 3.18 (2.54 – 3.93) | 1.53 (1.10 - 2.14) |
| March | 261 294 | 82 | 3.14 (2.50 – 3.90) | 1.51 (1.08 – 2.12) |
| April | 269 477 | 102 | 3.79 (3.09 – 4.59) | 1.82 (1.33 - 2.52) |
| May | 254 099 | 97 | 3.82 (3.10 – 4.66) | 1.84 (1.33 - 2.55) |
| June | 273 262 | 66 | 2.42 (1.87 – 3.07) | 1.16 (0.82 - 1.66) |
| July | 274 775 | 57 | 2.07 (1.57 – 2.69) | Ref |
| August | 289 237 | 98 | 3.39 (2.75 – 4.13) | 1.63 (1.18 - 2.26) |
| September | 287 003 | 110 | 3.83 (3.15 – 4.62) | 1.85 (1.34 - 2.54) |
| October | 300 222 | 118 | 3.93 (3.25 – 4.71) | 1.89 (1.38 - 2.60) |
| November | 299 048 | 115 | 3.85 (3.17 – 4.62) | 1.85 (1.35 - 2.55) |
| December | 297 432 | 97 | 3.26 (2.64 – 3.98) | 1.57 (1.13 - 2.18) |
| Total | 3 358 185 | 1 136 | 3.38 (3.19 – 3.59) |  |

|  |  |
| --- | --- |
